# Supplementary material for: Therapeutic potential of blocking GAPDH nitrosylation with CGP3466b in experimental autoimmune encephalomyelitis
Source: Front Neurol. 2023 Jan 24;13:979659. doi: 10.3389/fneur.2022.979659 (PMC9902867; doi:10.3389/fneur.2022.979659)
Supplement: Supplementary file 1 [file Data_Sheet_1.PDF]

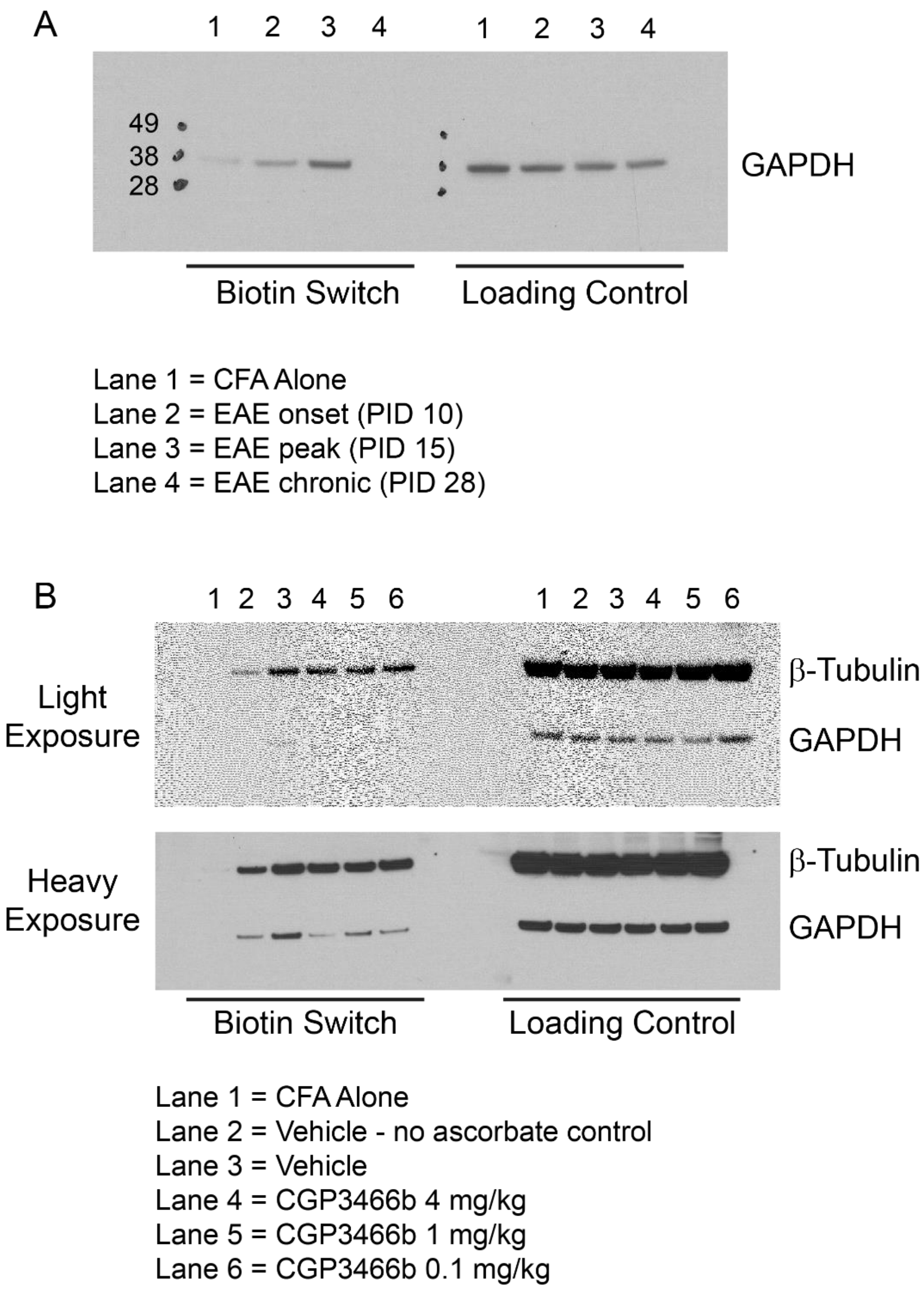

Supplementary Figure 1. Full immunoblots from Figure 1. **(A)** Full blot from Figure 1A. **(B)** Full blot from Figure 1B. For (B), biotin switch and loading control samples were run on different gels but exposed on same film, and blots were cut between molecular weight markers 38 and 49 kD to probe for GAPDH and β-Tubulin, respectively.

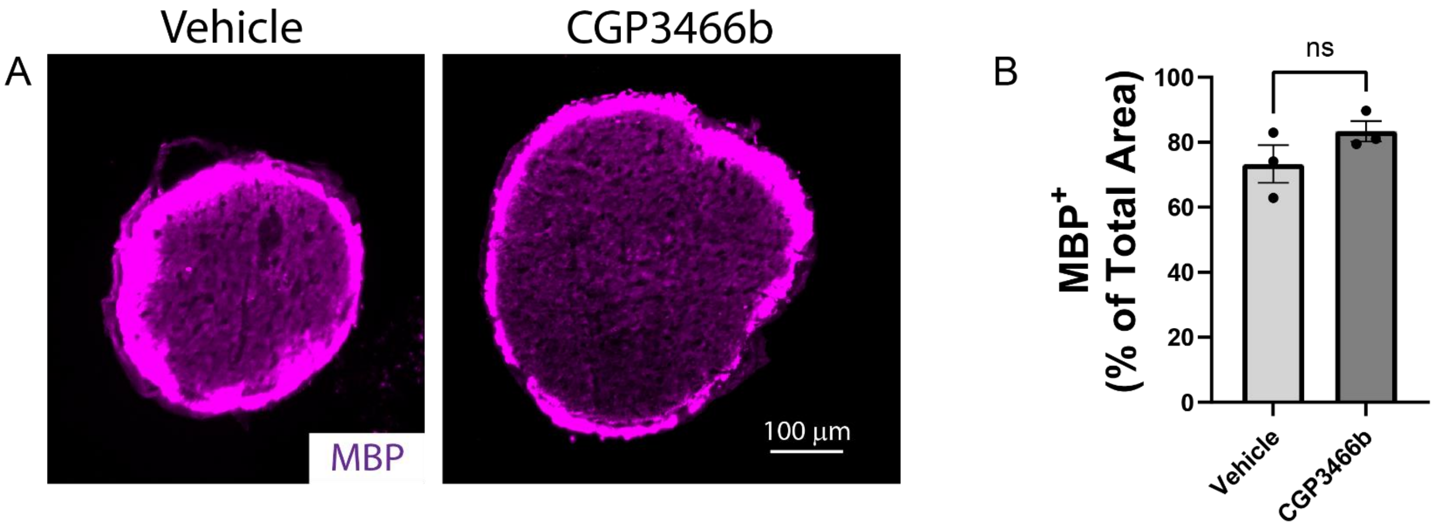

Supplementary Figure 2. Demyelination in the optic nerve following CGP3466b treatment. Mice were treated with vehicle or 4 mg/kg CGP3466b daily via i.p. injection starting on post-immunization day 0, and myelin basic protein (MBP) expression was analyzed by immunofluorescence staining of optic nerve at post-immunization day 28. The optic nerves included in the analysis correspond to the same cohort depicted in Figures 2D and 4B. **(A)** Representative image. **(B)** Quantification performed from n=3 mice per group.

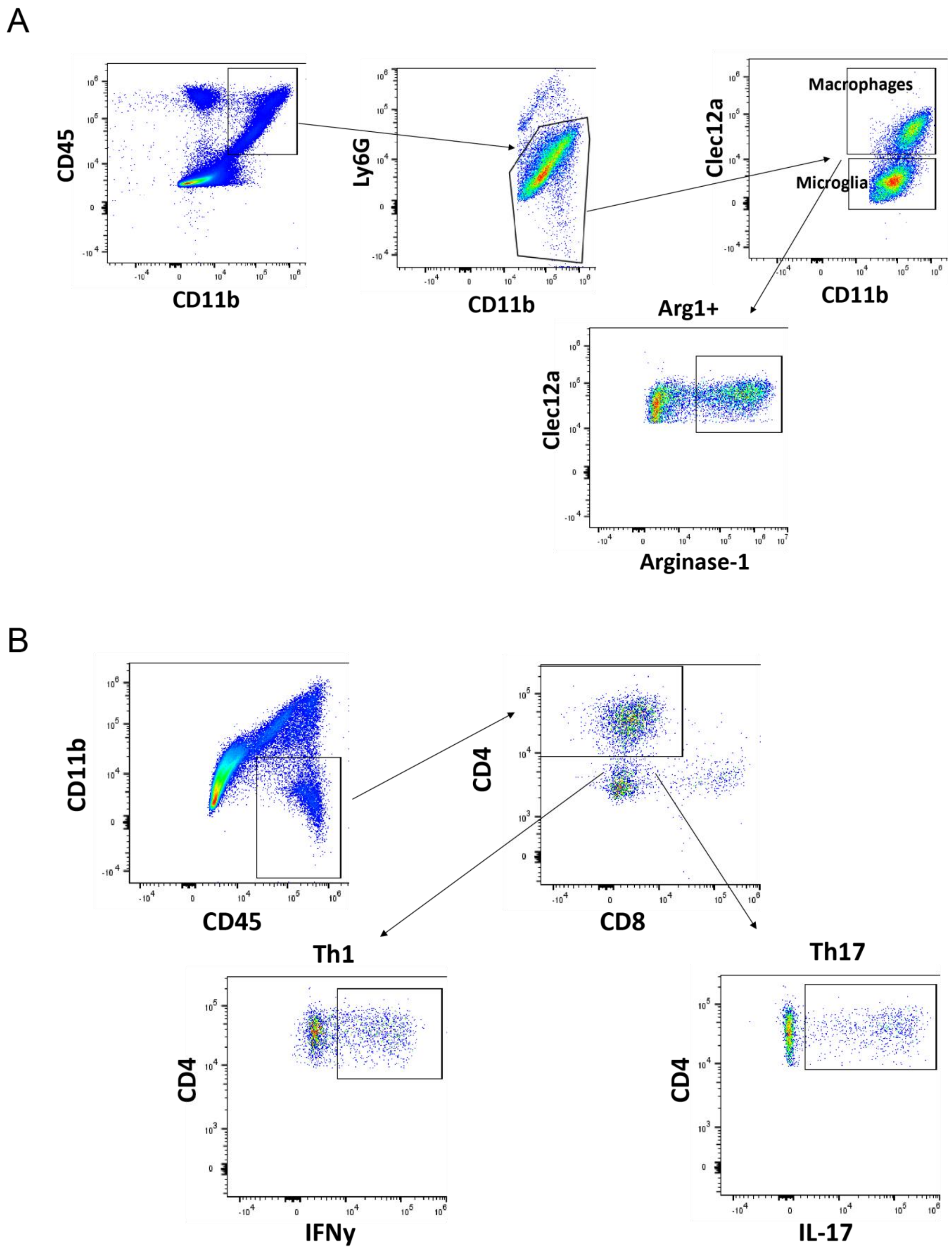

Supplementary Figure 3. Flow cytometry gating strategies for **(A)** microglia/macrophages and **(B)** CD4 cells. Corresponding results are shown in Figure 4.
